# The association between childhood trauma and suicidal ideation in medical students: the role of alexithymia and resilience

**DOI:** 10.3389/fpsyt.2025.1675266

**Published:** 2025-10-09

**Authors:** Xiaomei Gao, Siqi Mu, Daofen Zhang, Ping Li, Wanrong Wang, Xinyang Hu, Peng Wang

**Affiliations:** ^1^ The First School of Clinical Medicine, Anhui Medical University, Hefei, Anhui, China; ^2^ Department of Endocrinology, Sir Run Run Shaw Hospital, Zhejiang University School of Medicine, Hangzhou, Zhejiang, China; ^3^ Department of Respiratory and Critical Care Medicine, The First Affiliated Hospital of Anhui Medical University, Hefei, China; ^4^ School of Nursing, Anhui Medical University, Hefei, Anhui, China

**Keywords:** childhood trauma, suicidal ideation, alexithymia, resilience, medical students

## Abstract

**Objective:**

To reveal the association between childhood trauma and suicidal ideation in medical students and explore the potential mediating roles of alexithymia and psychological resilience.

**Methods:**

Based on a cross-sectional survey conducted at a medical university in Anhui Province, 2,377 medical students were included. Assessments were performed using the Childhood Trauma Questionnaire, the Toronto Alexithymia Scale, the Resilience Scale, and the Suicidal Ideation Scale.

**Results:**

Our results showed that childhood trauma significantly increased the risk of suicidal ideation in medical students (β=0.500, 95% CI: [0.470, 0.540]; The association was mediated by an alexithymia-resilience chain (mediating effect β=0.03, 95% CI: [0.029,0.040].

**Conclusion:**

Emphasizing attention to medical students’ childhood trauma experiences, focusing on enhancing their emotion-processing abilities, and promoting psychological resilience represent effective strategies for preventing suicide risk in this population.

## 1 Introduction

Suicide among university students has become a major challenge in the realms of campus public health and safety due to its unpredictability. Medical students face unique professional and occupational demands and their mental health is crucial for individual growth and development. Medical students showed more severe anxiety, depression, and obsessive-compulsive tendencies compared to the average levels among university students in other disciplines (1). Academic stress is prevalent among contemporary college students; however, medical students are confronted with a unique set of severe long-term stressors. These stressors cumulate and interact with one another, and compared to their peers in other majors, this cumulative effect significantly elevates the risk of adverse mental health outcomes in medical students. First of all, medical students have a heavier academic burden, they must master a large amount of complex scientific and clinical knowledge, which is directly related to their future career prospects, and there are many high-risk exam pressures (2). Second, intense coursework, clinical rotations, and self-study schedules inevitably lead to long-term sleep deprivation, which directly impairs emotion regulation, cognitive function, and coping mechanisms, and exacerbates vulnerability to stress and psychopathology (3). Third, medical students are prematurely and repeatedly exposed to human suffering, death, and moral dilemmas during their clinical training, leading to particular emotional distress that may contribute to internal stress and emotional depression (4).

Suicidal ideation refers to thoughts or cognitions about ending one’s own life and is a necessary psychological precursor to suicidal behavior. Over the past decade, the prevalence of suicidal ideation among Chinese medical students has been approximately 13% (5). A latent profile analysis of suicide risk factors in university students revealed that, in terms of individual vulnerability factors, borderline personality traits, deficits in emotional regulation ability, childhood trauma, impulsive personality traits, negative coping with stress, and psychological resilience constitute risk factors for suicidal ideation (6).

Childhood trauma refers to any form of abuse and neglect experienced during an individual childhood development (7). As an early-life environmental factor, childhood trauma serves as an important predictor for numerous psychiatric disorders (8). Studies indicated that childhood trauma was associated with a substantially increased risk of adolescent suicide and suicidal ideation (9). Suicidal behavior is linked to an individual inherent diathesis and exposure to traumatic events. Childhood traumatic experiences not only represent persistent underlying factors but also constrain the development of psychological resilience. Severe trauma may precipitate alterations in personality structure, ultimately elevating suicide risk in adulthood (10). This study aims to investigate the impact of childhood trauma on suicidal ideation among medical students and elucidate its underlying mechanisms.

Alexithymia, also referred to as “Emotion Articulation Disorder” or “Affective Expressiveness Impairment,” denotes an individual’s impairment in identifying others’ emotions and expressing their own emotions. It manifests primarily as difficulties in emotion recognition, emotion expression, and externally oriented thinking (11). Research on the formation mechanisms of alexithymia indicates that its development correlates with adverse childhood experiences. It arises from disruptive events and relationships that inhibit the development of emotional functioning during early childhood (12), with subsequent reinforcement through sociocultural and relational contexts. Alexithymia is recognized as an impairment in emotion cognition, processing, and regulation (13). Concurrently, an individual’s level of alexithymia is closely associated with physical and psychological symptoms. Due to deficits in emotional cognitive regulation and maladaptive emotion management strategies, individuals with alexithymia tend to employ dysfunctional defense mechanisms when confronting negative events. This increases their susceptibility to psychological problems, including suicidal ideation and behavior, exerting detrimental effects on physical and mental health (14, 15). Based on this evidence, it can be hypothesized that alexithymia may mediate the relationship between childhood trauma and suicidal ideation in university students.

In real-world contexts, however, not all individuals with childhood trauma histories develop severe psychological disturbances. Luthar et al. proposed that protective factors that enable individuals to cope with adverse environments or crises can promote positive psychological development among trauma-exposed children—one such factor being psychological resilience (16). Richardson’s (2002) Dynamic Model of Resilience posits that the interaction between protective and risk factors drives individuals through cycles of balance, imbalance, and rebalance across physiological, psychological, and social domains—a process that underlies the development of psychological resilience. Childhood trauma, as a chronic stressor, disrupts protective and adaptive systems, thereby hindering the development of psychological resilience (17). Studies of children, adolescents, and university students consistently demonstrate that childhood trauma negatively predicts an individual’s level of psychological resilience and impairs its development (18, 19). As a buffering protective mechanism for mental health, psychological resilience mediates or moderates the relationship between negative life events and psychological problems such as depression (20, 21). Sher’s research identified psychological resilience as a critical protective factor against suicide-related psychological behaviors (22), while Stark et al. found it negatively predicts suicidal ideation in adolescents (23).

Alexithymia prevents individuals from effectively managing the negative emotional states resulting from childhood trauma. The inability to recognize and regulate painful emotions consumes cognitive resources that could otherwise be used for adaptive coping and problem-solving, thereby diminishing the capacity for psychological resilience (24). Alexithymia is characterized by deficits in identifying and describing one’s own emotions, and it also impairs empathy and the ability to interpret the emotional states of others. This makes it difficult to form and maintain close, supportive interpersonal relationships. As a result, individuals may lack the key social buffers that promote psychological resilience (25). Individuals who have experienced childhood trauma often develop alexithymia as a defense mechanism to avoid the unbearable pain. This emotional processing defect directly weakens the foundation of psychological resilience through multiple pathways (26). Alexithymia is not only a related factor but also an important mechanism that partly explains how early trauma leads to the continuous weakening of resilience. Consequently, higher levels of psychological resilience facilitate maintenance of healthy psychological states and reduce the incidence of suicide-related behaviors. Thus, this study will examine the role of psychological resilience in the association between childhood trauma and suicidal ideation among medical students.

Emotion management ability also constitutes a significant factor in the development of an individual’s psychological resilience. Research indicates that individuals with proficient emotion management abilities attain higher levels of psychological resilience (27). Conversely, alexithymia—characterized primarily by deficits in emotion-related cognition and regulation functions—impedes the development of psychological resilience. In their investigation of the relationship between alexithymia and psychological resilience, Craparo et al. found significant negative correlations between alexithymia and all dimensions of psychological resilience (28). Similarly, Morice et al. identified a negative correlation between alexithymia and psychological resilience when examining the predictive role of alexithymia, further demonstrating that lowering alexithymia levels enhances psychological resilience (29). Research indicates that affective temperaments constitute a genetic factor in mood disorders. These emotional dispositions contribute to complex conditions such as psychiatric syndromes, personality disorders, and suicidal behavior, while also influencing disease progression and treatment adherence (30). Consequently, personality and affective temperament may also serve as precipitating factors for suicide, warranting further investigation.

Therefore, this study will first investigate whether alexithymia and psychological resilience act as a chained mediating pathway between childhood trauma and suicidal ideation in university students. On this basis, we hypothesized that alexithymia and psychological resilience would sequentially mediate the relationship between childhood trauma and suicidal ideation among medical students.

## 2 Research methods

### 2.1 Participants

This study employed random sampling to recruit medical students from a medical university in Anhui Province as participants. Scale assessments were administered through group testing. We selected all undergraduate students majoring in clinical medicine, anesthesiology and nursing from the first to the fifth year of a medical university. Stratification was conducted based on medical specialties and grades, resulting in a total of 15 strata. We obtained the list of all students’ student numbers, names, specialties, grades, and genders from the university’s academic affairs office. The margin of error was set at 5% and the confidence level was set at 95%. Following the exclusion of incomplete questionnaires, 2,377 valid responses were retained (1,176 males, 1,201 females; mean age = 19.03 ± 1.33 years). All participants were fully informed about the study’s purpose and significance and provided written informed consent prior to participation. The research protocol was approved by the institutional ethics committee of the first author’s affiliated university. Students identified with high suicidal ideation through screening received follow-up interventions conducted jointly by clinical experts from the research team and psychological counselors at the university’s student mental health center.

### 2.2 Instruments

#### 2.2.1 Childhood trauma questionnaire​​

Childhood trauma was assessed using the Childhood Trauma Questionnaire-Short Form (CTQ-SF), originally developed by Bernstein et al. (1998). The Chinese version validated by Zhao Xingfu et al. was used. The 28-item scale evaluates five clinical subtypes of maltreatment: Emotional Abuse, Physical Abuse, Sexual Abuse, Emotional Neglect, and Physical Neglect. Participants scoring in the ‘Low’ to ‘Severe’ range on any subscale were considered to have a positive history of that specific type of childhood maltreatment (31). Each subscale contains 5 items rated on a 5-point Likert scale ranging from 1 (never) to 5 (very often). In this study, we tallied the total score of the scale. A higher score indicates more severe childhood trauma. The Cronbach’s α coefficient was 0.720 in this study, indicating good reliability and validity.

#### 2.2.2 Toronto alexithymia scale​​

Alexithymia was assessed using the 20-item Toronto Alexithymia Scale (TAS-20). The instrument comprises three dimensions: Difficulty Identifying Feelings (DIF), Difficulty Describing Feelings (DDF), and Externally Oriented Thinking (EOT). Items are rated on a 5-point Likert scale ranging from 1 (strongly disagree) to 5 (strongly agree). The Cronbach’s α coefficient was 0.726 in this study, indicating adequate reliability and validity.

#### 2.2.3 Positive and negative suicide ideation inventory

Suicidal ideation was measured using the Positive and Negative Suicide Ideation inventory (PANSI), translated and revised by Wang Xuezhi et al. (2011). This 14-item scale employs a 5-point response format from 1 (“never like this”) to 5 (“always like this”). The PANSI contains two subscales: Positive Suicide Ideation (6 items, reverse-scored) and Negative Suicide Ideation (8 items, forward-scored). Higher total scores indicate greater severity of suicidal ideation. The Cronbach’s α coefficient was 0.902 in this study, demonstrating excellent reliability and validity.

#### 2.2.4 Connor-Davidson Resilience Scale (CD-RISC-10)

Psychological resilience was measured using the 10-item Connor-Davidson Resilience Scale (CD-RISC-10), abbreviated by Campbell-Sills et al. from the original 25-item CD-RISC developed by Connor and Davidson (2003). Items are rated on a 5-point scale ranging from 0 (never) to 4 (very often), with higher total scores indicating better psychological resilience. The scale demonstrated excellent reliability in this study (Cronbach’s α = 0.961).

### 2.3 Intervention programs for high-risk groups

Participants with a high risk of suicide determined by scale scores will be followed up by phone by our team’s well-trained psychologists, who will provide them with moderate psychological education and strongly recommend that they seek professional help. We will also help them contact the university’s mental health services and continue to maintain follow-up contact to ensure that they successfully receive professional services.

### 2.4 Statistical analysis

Statistical analyses were performed using SPSS 21.0. Descriptive statistics characterized continuous normally distributed variables as mean ± standard deviation. Pearson correlation analysis examined inter-variable relationships. A chained mediation model was tested using Hayes’ PROCESS macro version 3.3. The significance level was set at α = 0.05 (two-tailed).

## 3 Results

### 3.1 Descriptive statistics and correlation analysis of variables​​

Descriptive statistics and correlation analyses were performed for all variables. Results indicated that: Childhood trauma, alexithymia, and suicidal ideation were significantly positively correlated between each pair of variables; Psychological resilience showed significant negative correlations with childhood trauma, alexithymia, and suicidal ideation (**Table 1**).

**Table 1:** The correlation between mental health indicators in medical students.

	Childhood trauma	Alexithymia	Resilience	Suicidal ideation
Childhood Trauma	1			
Alexithymia	0.265***	1		
Resilience	-0.164***	-0.419***	1	
Suicidal ideation	0.503***	0.432***	-0.467***	1

***P<0.001.

### 3.2 Mediation effects of alexithymia and psychological resilience​​

Mediation analyses were conducted using PROCESS 3.3 in SPSS, with gender and age as covariates, to test the bootstrap-based mediating effects of alexithymia and psychological resilience on the relationship between childhood trauma and suicidal ideation. Regression results (**Table 2**) demonstrated that: After introducing mediators, childhood trauma exerted a significant direct effect on suicidal ideation (β=0.400, 95% CI [0.370, 0.430]), accounting for 80% of the total effect. Childhood trauma positively predicted alexithymia (β=0.260 [0.220, 0.300], p <0.001) and negatively predicted psychological resilience (β = -0.06 [-0.100, -0.020], p < 0.01). Alexithymia negatively predicted psychological resilience (β = -0.410 [-0.440, -0.370], p < 0.001) and positively predicted suicidal ideation (β = 0.200 [0.160, 0.230] p < 0.001). Psychological resilience negatively predicted suicidal ideation (β = -0.320 [-0.350, -0.290], p < 0.001).

**Table 2:** The regression analysis.

Outcome	Predictors	β with 95% CI	P value	Standardized coefficients
Suicidal ideation (Y)	Gender	0.01[-0.02, 0.05]	0.386	0.02
	Age	0.01[0.002, 0.02]	0.016	0.04
	Childhood Trauma	0.75[0.69, 0.80]	<0.001	0.50
Alexithymia (M1)	Gender	-0.08[-0.12, -0.03]	0.0007	-0.07
	Age	-0.01[-0.02, 0.001]	0.083	-0.03
	Childhood Trauma	0.48[0.41, 0.55]	<0.001	0.26
Resilience (M2)	Gender	-0.04[-0.01, 0.03]	0.245	-0.02
	Age	-0.01[-0.03, 0.006]	0.219	-0.02
	Childhood Trauma	-0.16[-0.26, -0.06]	0.002	-0.06
	Alexithymia	-0.59[-0.65, -0.54]	<0.001	-0.41
Suicidal ideation (Y)				
	Gender	0.03[-0.0001, 0.06]	0.051	0.03
	Age	0.01[0.004, 0.02]	0.003	0.05
	Childhood Trauma	0.59[0.55, 0.64]	<0.001	0.40
	Alexithymia	0.16[0.13, 0.19]	<0.001	0.20
	Resilience	-0.18[-0.20, -0.16]	<0.001	-0.32

**Figure 1:**
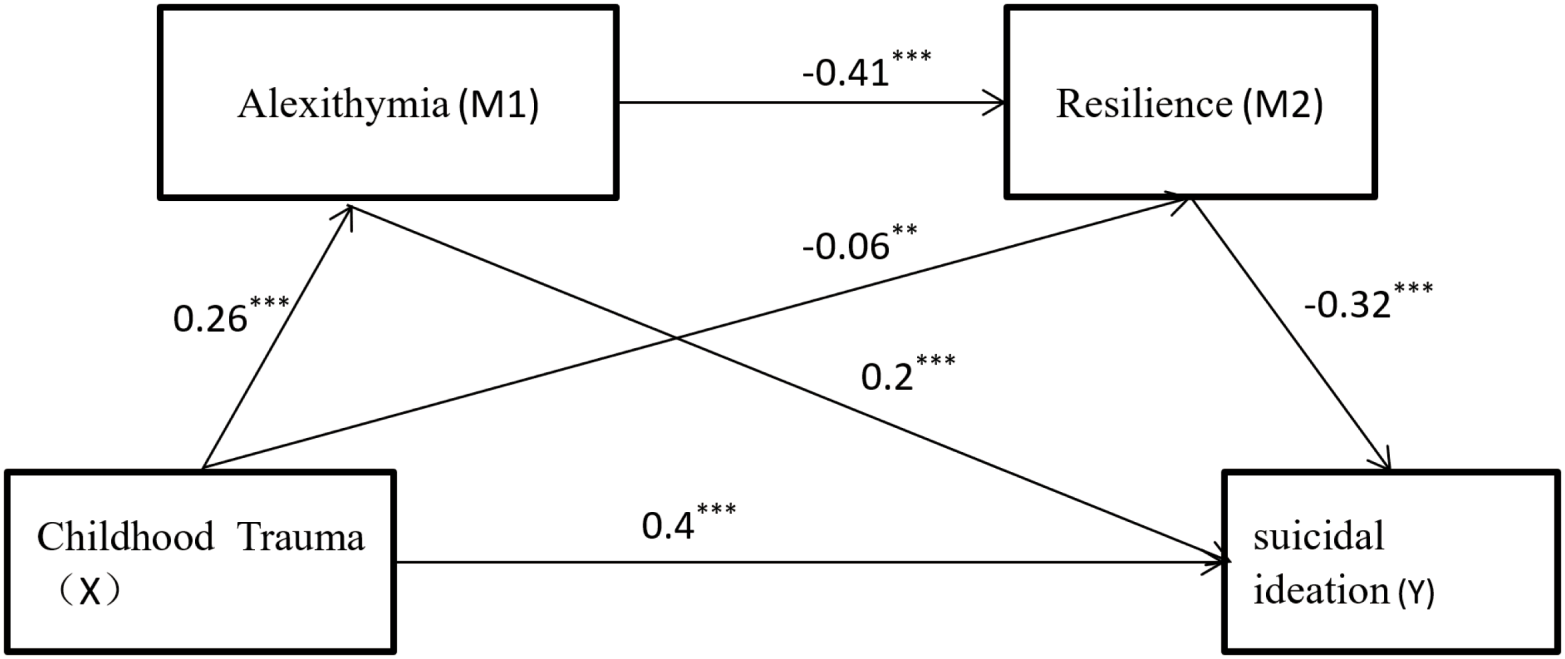
Mediating effect diagram of alexithymia and psychological resilience. ***P < 0.001.

Mediation analysis with 5,000 bootstrap resamples estimated 95% confidence intervals (see **Table 3** and **Figure 1**). Results indicated a total indirect effect of childhood trauma on suicidal ideation of 0.10, accounting for 20% of the total effect (0.50). This mediation comprised three significant pathways: Path 1: Childhood trauma → Alexithymia → Suicidal ideation (Indirect effect = 0.05, 10% of total effect). Path 2: Childhood trauma → Psychological resilience → Suicidal ideation (Indirect effect = 0.02, 4% of total effect). Path 3: Childhood trauma → Alexithymia → Psychological resilience → Suicidal ideation (Indirect effect = 0.03, 6% of total effect). All pathways showed statistically significant mediation (95% CIs excluded zero). In addition, we conducted a sensitivity analysis stratified by gender to further validate the robustness of the mediation pathway (**Supplementary Materials**). The results demonstrate that childhood trauma not only directly predicts suicidal ideation in university students but concurrently exerts significant indirect predictive effects through the partial mediation of alexithymia or psychological resilience and the chained mediation effect of alexithymia and psychological resilience.

**Table 3:** The mediating role of alexithymia and resilience in the relationship between childhood trauma and suicidal ideation.

Mediating pathway	Effect size with 95% CI	Proportion of mediating effect
Total direct effect	0.100[0.080,0.130]	20%
Alexithymia	0.050[0.036,0.070]	10%
Resilience	0.020[0.003,0.040]	4%
Alexithymia and Resilience	0.030[0.029,0.040]	6%

Due to the cross-sectional design of this study and reliance on self-assessment scale data, it may introduce selection bias, recall bias, sampling bias. We have taken corresponding measures to address these biases, for example, statistical test power, scale validity testing, and rigorously designed sampling standards, to achieve good control effects.

## 4 Discussion

This study elucidates the impact of childhood trauma on suicidal ideation in medical students and its underlying mechanisms. Correlation analyses demonstrated significant positive intercorrelations among childhood trauma, alexithymia, and suicidal ideation, while all three variables showed significant negative correlations with psychological resilience. Regression analyses revealed that after accounting for mediators, childhood trauma maintained a significant direct positive prediction of suicidal ideation. The significant direct effect of childhood trauma on suicidal ideation suggests the existence of other key mechanistic pathways. Future research should prioritize investigating neurobiological dysregulation (e.g., altered stress response in the HPA axis, epigenetic changes, brain structural abnormalities) and psychosocial factors (e.g., prolonged social isolation, fatigue, or frustrated academic achievement) as potential mediating variables (32, 33). Integrating multi-level data, including biomarker testing and detailed social network analysis, is crucial to uncovering the complex etiology of suicide risk in individuals with childhood trauma. Simultaneously, both alexithymia and psychological resilience functioned as partial mediators in the relationship between childhood trauma and suicidal ideation, while they additionally operating through a chained mediation pathway whereby childhood trauma influenced suicidal ideation via alexithymia’s impact on psychological resilience. These findings collectively establish alexithymia and psychological resilience as critical bridging factors linking childhood trauma to suicidal ideation in medical students.

Childhood trauma influences medical students’ suicidal ideation through the mediating role of alexithymia. According to the Emotion Processing Model, individuals with a history of childhood trauma tend to adopt avoidant emotion-processing strategies to avoid overwhelming negative emotions triggered by traumatic experiences. While such strategies may provide short-term relief from distress, chronic avoidance can lead to unconscious emotional disengagement and suppression of affect. This process impedes the ability to identify internal emotional states and describe feelings accurately, instead promoting externally oriented thinking, which are the core features of alexithymia (34). Previous studies confirm that childhood trauma functions as a chronic stressor and constitutes a significant etiological factor for alexithymia (35). Alexithymia subsequently increases susceptibility to psychological disorders and maladaptive behaviors (36), with elevated suicide risk (13). Aligned with our findings, medical students exposed to childhood trauma exhibit heightened alexithymia. This emotional avoidance prevents adequate processing of trauma-related negative emotions, thereby amplifying suicidal ideation through blocked emotional expression and feedback.

Our study identified psychological resilience as a significant mediator in the relationship between childhood trauma and suicidal ideation among medical students. Specifically, individuals with traumatic histories exhibit reduced psychological resilience, which in turn increases vulnerability to suicidal ideation. This finding aligns with established research demonstrating that psychological resilience functions as a protective factor capable of mitigating the adverse effects of negative life events (37) and reducing susceptibility to suicidal ideation (18) while enhancing adaptive functioning. Concurrently, childhood trauma negatively impacts the development of psychological resilience (19, 38). Chronic exposure to trauma fosters maladaptive cognitive patterns wherein individuals increasingly interpret adverse events through negative schemas, gradually internalizing dysfunctional self-perceptions. This process diminishes self-confidence during stressful encounters, promotes passive coping strategies, and ultimately compromises resilience capacity, thereby precipitating mental health deterioration. These findings suggest that psychological resilience may play a crucial mediating role in the pathway linking childhood trauma to suicidal ideation among medical students.

Mediation analysis revealed that childhood trauma additionally influences medical students’ suicidal ideation through the sequential mediating pathway of alexithymia and psychological resilience. Individuals with alexithymia experience chronic impairment in identifying, describing, and processing emotional states, preventing timely resolution of psychological distress. Prolonged emotional dysregulation may induce sensitization effects, thereby diminishing psychological resilience (39) and eventually increase vulnerability to suicidal ideation (13). These findings corroborate Martin and Pihl’s (1985) stress-alexithymia hypothesis (40), which posits that deficient emotional self-awareness—manifested as confusion or inability to recognize one’s emotions—compromises stressor identification and impedes adaptive coping. Consequently, alexithymic individuals exhibit maladaptive responses to adverse events, characterized by reduced implementation of active strategies such as social support seeking (41, 42). Thus, childhood trauma predisposes individuals to alexithymia, which subsequently impairs resilience development, promotes passive coping strategies during adversities, and ultimately elevates risk for suicide-related outcomes including suicidal ideation. In addition, the mechanism of how affective temperament affects suicidal ideation and resilience needs to be further explored, and its mediating or moderating effect on the onset of affective disorders needs to be further explored (30). Incorporating the assessment of temperament traits into the multidimensional psychiatric diagnostic process can optimize treatment and prognosis estimation.

It is worth noting that this study was carried out in a specific socio-cultural context in China, especially for the Chinese medical student population. China has a unique cultural background, such as an emphasis on academic achievement, filial piety, and a special attitude towards mental health and suicide. These factors may affect the manifestation of childhood trauma, the expression of alexithymia, and the formation of suicidal ideation. Therefore, the strength of association observed in our model and the proposed mediating paths may not be directly generalized to Western cultures or other different cultural contexts. In addition, on sensitive topics such as childhood trauma, the self-reports of the study subjects are affected by recall bias. At the same time, this paper uses a cross-sectional study, which cannot make any definite causal inference about the relationship between variables. Therefore, we have avoided making statements about causality in the text to enhance the rigor of the article.

Finally, we propose corresponding limitations and future research directions. For example, longitudinal design is used to determine time series and causal relationships, combined with multiple methods to evaluate results, reduce dependence on self-reported data and recall bias, conduct cross-cultural reproduction studies, and test the universality of the model. In the future, experiments can also be conducted to test the hypothesis that reducing alexithymia and enhancing psychological toughness can effectively reduce the risk of suicide in students with a history of childhood trauma. Furthermore, future research could inform the development of targeted interventions to address emotional processing deficits and emotional dysregulation—such as mindfulness-based stress reduction (MBSR) and emotion-focused therapy. Structured intervention programs could include resilience training, cognitive behavioral therapy (CBT), and the promotion of peer support groups within medical schools. These interventions should be integrated into medical school curricula to proactively address their psychological needs.

## 5 Conclusion

This cross-sectional study demonstrates that greater exposure to childhood trauma is significantly associated with an elevated risk of suicidal ideation among adolescents. Specifically, alexithymia and resilience may function as critical mediating factors in the pathway linking childhood trauma to suicidal ideation. Psychological resilience can be enhanced through targeted interventions targeting its key constituent components, thereby mitigating suicidal ideation among medical students.

## Data Availability

The original contributions presented in the study are included in the article/**Supplementary Material**. Further inquiries can be directed to the corresponding author.
