# Supplementary material for: The association between childhood trauma and suicidal ideation in medical students: the role of alexithymia and resilience
Source: Front Psychiatry. 2025 Oct 9;16:1675266. doi: 10.3389/fpsyt.2025.1675266 (PMC12546031; doi:10.3389/fpsyt.2025.1675266)
Supplement: Supplementary file 1 [file DataSheet1.pdf]

Supplementary materials

Sensitivity analyses stratifying by gender (male)

Table 1. The mediating role of alexithymia and resilience in the relationship between childhood trauma and suicidal ideation

Mediating pathway	Effect size with 95% CI	Proportion of mediating effect
Total indirect effect	0.088 [0.060,0.120]	16.57%
Alexithymia	0.059 [0.037,0.081]	10.99%
Resilience	0.010 [-0.07,0.030]	1.82%
Alexithymia and Resilience	0.020 [0.013,0.030]	3.76%

Sensitivity analyses stratifying by gender (female)

Table 2. The mediating role of alexithymia and resilience in the relationship between childhood trauma and suicidal ideation

Mediating pathway	Effect size with 95% CI	Proportion of mediating effect
Total indirect effect	0.147 [0.11,0.20]	31.28%
Alexithymia	0.033 [0.013,0.06]	7.02%
Resilience	0.047 [0.02,0.08]	10%
Alexithymia and Resilience	0.067 [0.05,0.09]	14.26%
